# A simple method to evaluate the number of bradyrhizobia on soybean seeds and its implication on inoculant quality control

**DOI:** 10.1186/2191-0855-1-21

**Published:** 2011-07-19

**Authors:** Claudio Penna, Rosana Massa, Florencia Olivieri, Gabriel Gutkind, Fabricio Cassán

**Affiliations:** 1 Laboratorio de Investigación y Desarrollo Básico. Merck Crop Bioscience Argentina. Buenos Aires, Argentina; 2 Departamento de Microbiología, Inmunología y Biotecnología, Facultad de Farmacia y Bioquímica, Universidad de Buenos Aires, Argentina; 3 Laboratorio de Fisiología Vegetal, Universidad Nacional de Rio Cuarto, Córdoba, Argentina

**Keywords:** *Bradyrhizobium japonicum*, inoculants, soybean, quality control

## Abstract

Soybean seeds are non-sterile and their bacterial population interferes with the enumeration of beneficial bacteria, making it difficult to assess survival under different conditions. Within this context, the principal aims of this work were: (1) to improve a selective media for the enumeration of *B. japonicum *recovered from inoculated soybean seeds; (2) to establish the most representative mathematical function for *B. japonicum *mortality on soybean seeds after inoculation; (3) to evaluate if environmental or physiological conditions modify *B. japonicum *mortality on soybean seeds; and (4) to create a new protocol for quality control of soybean inoculants. We successfully evaluated the combination of pentachloronitrobenzene and vancomycin added to the yeast-mannitol medium to inhibit most fungi and Gram-positive soybean microbiota, thus producing reliable counts of *B. japonicum *from inoculated soybean seeds. Percentages of recovery and survival factors were obtained and used to construct a two-phase exponential decay non-linear regression function. High temperature and desiccation decreased these parameters, while the optimization of temperature and the use of osmoprotective compounds with inoculants increased them. The use of this protocol minimized heterogeneity between experiments and may be considered more reliable than the simple expression of direct colony count of bacteria recovered from seeds.

## Introduction

Soybeans [*Glycine max *L. (Merr.)] are one of the major crops in grain-producing countries worldwide. Particularly in Argentina, production for 2010 has been estimated in about 50.0 million tons, which represents 7 percent less than the record of 54.0 million tons covering a production area of 18.0 million hectares (USDA, 2010). Improved crop productivity has been associated with different practices, such as the incorporation of agronomical, physical, chemical or biological technologies. The use of mineral fertilizers has been subordinated to biological fertilization, principally due to its economical and environmental advantages (Adesemoye and Kloepper, 2009). Inoculation with symbiotic nitrogen-fixing *Bradyrhizobiaceae *bacteria has become a simple and effective way to significantly improve soybean yield and productivity. Within the genus *Bradyrhizobium, B. japonicum *is the best example of successful symbiotic fixation under very large scale field conditions. Therefore, *B. japonicum *has been successfully incorporated as the active principle of soybean inoculants in Argentina, Brazil, Paraguay, USA, Canada and other producing countries worldwide in the last 30 years.

Inoculant improvement has been associated to more effective methods to evaluate the quality of bacterial formulations (inoculants) (Tong and Sadowsky, 1994), as well as those related with the isolation, identification and enumeration of bacteria in carriers, seeds, soil and plants. Thus, the enumeration of *B. japonicum *could be considered the most essential method used for defining the soybean inoculants quality, even before the evaluation of activity under experimental field conditions.

Currently *B. japonicum *enumeration in liquid formulation currently includes several methods; however, the most probable-number plant infection method (MPN) proposed by Vincent (1970), and the direct colony-forming unit (CFU) counting on agar plates proposed by Somasegaran and Hoben (1994) are still the most frequently used methods in several laboratories worldwide.

Several discrepancies between viable counts and the field efficacy of the inoculants have been reviewed by Catroux et al. (2001). Results as cfu.ml^-1 ^counting only represent the theoretical maximum provided by the inoculants under field conditions. Some sub-lethally damaged bacteria may survive under optimal growth conditions (e.g. culture medium), but not on seed or soil. On the other hand, the agronomical behavior of soybean inoculants under field conditions show a solid tendency in different reports that lead to assume that "when the number of viable rhizobia by seed increases, nodulation and yield are improved", as proposed by Brockwell et al. (1988). According to Weaver and Frederick (1972) more than 10^5 ^*B. japonicum *cells provided per seed are required to obtain the maximum number of nodules on the tap root. Similarly, Obaton and Rollier (1970) reported an increase in soybean yield in field conditions when the inoculation rate increased to 10^6 ^bacteria inoculated per seed. In other experiments, Hume and Blair (1992) reported that the effect on soybean yield could vary by the dilution of the same inoculant or the inoculation with different number of *B. japonicum *cells.

Therefore, both the cell number and the bacterial physiological state could be responsible for improving inoculant functionality and soybean productivity in agronomic conditions. Despite this fact there are no published reports that correlate a mathematical function with *B. japonicum *death from inoculated soybean seeds. For that reason, it is mandatory to find a more trustworthy reproducible method, than direct cell counting and simpler than the MPN plant assay, which would allow a more consistent way to evaluate bradyrhizobia cell numbers after inoculation. In this direction, the number of viable bradyrhizobia provided per seed counted on selective media agar plates could be considered the best method. However, its use may be impaired because soybean seeds under actual field growing conditions are obviously non-sterile and the bacterial or fungal seed microbiota normally interferes with the counting, which implies that bradyrhizobia cell enumeration is normally more difficult starting from non-sterile seeds than from inoculants during quality control procedures (Hiltbold et al., 1980).

Our hypothesis proposes that *Bradyrhizobium *cells applied on soybean seeds during inoculation, die according to a mathematical function, and this phenomenon plus the selection of specific culture conditions could be useful to establish a trustworthy counting method for bradyrhizobia from seeds, and to design a new protocol for inoculant quality control. The aims of this work were: (1) to improve a selective media for the enumeration of *B. japonicum *obtained from inoculated soybean seeds; (2) to establish the most representative mathematical function for *B. japonicum *mortality on soybean seeds after inoculation; (3) to evaluate if some environmental or physiological conditions may modify *B. japonicum *mortality or survival capacity on soybean seeds; and (4) to propose a reliable method for quality control evaluation of soybean inoculants.

## Materials and methods

### Microorganisms

Table 1 enumerates the 16 strains used in this work that belong to different species with a well-documented history of use as rhizobial inoculants in the Americas. They were obtained either from R. Stewart Smith from Merck Crop Bioscience, Winsconsin, USA (currently deposited in CCM-A WDCM29, Colección de Cultivos Microbianos, Facultad de Farmacia y Bioquimica, Universidad de Buenos Aires, Argentina) or provided by IMYZA-WDCM31, Instituto de Microbiología y Zoología Agrícola, Castelar, Argentina.

**Table 1:** Minimal inhibitory concentration (MIC) of 16 *Rhizobiales *strains

Scientific nomenclature	Strain	Origin	**MIC (μg.ml**^**-1**^**)**^(*)^
*Rhizobium leguminosarum *biovar viceae	D36	WDCM31^(1)^	>16
*Rhizobium leguminosarum *biovar viceae	175G10b	Merck Crop Bioscience^(2)^	2
*Rhizobium leguminosarum *biovar trifolii	162BB1	WDCM31^(1)^	4
*Rhizobium leguminosarum *biovar trifolii	162P17	WDCM31^(1)^	2
*Bradyrhizobium japonicum*	E109	WDCM31^(1)^	>16
*Bradyrhizobium japonicum*	61A227	Merck Crop Bioscience^(2)^	>16
*Bradyrhizobium japonicum*	61A228	Merck Crop Bioscience^(2)^	>16
*Bradyrhizobium japonicum*	61A273	Merck Crop Bioscience^(2)^	>16
*Bradyrhizobium *spp.	8A57	Merck Crop Bioscience^(2)^	>16
*Mesorhizobium huakii*	LL32	WDCM31^(1)^	4
*Rhizobium loti*	95C14	Merck Crop Bioscience^(2)^	16
*Rhizobium loti*	95C11	Merck Crop Bioscience^(2)^	4
*Sinorhizobium meliloti*	B401	WDCM31^(1)^	>16
*Sinorhizobium meliloti*	102F77b	Merck Crop Bioscience^(2)^	16
*Sinorhizobium meliloti*	102F51a	Merck Crop Bioscience^(2)^	8
*Sinorhizobium meliloti*	102F34a	Merck Crop Bioscience^(2)^	16

Bacterial nomenclature, origin and vancomycin minimal inhibitory concentration (MIC) corresponding to 16 *Rhizobiales *strains selected for this work. The MIC methodology was performed according to Clinical and Laboratory Standards Institute (CLSI) recommendations.

^(*) ^Minimal Inhibitory Concentration (MIC) for vamcomycin

^(1) ^Corresponding to IMYZA-Instituto de Microbiología y Zoología Agrícola. Castelar. Argentina

^(2) ^Currently deposited in CCM-A WDCM29

### Formulation and evaluation of the selective medium

The standard selective medium Yeast Extract-Mannitol (YEM) proposed by Vincent et al. (1970) and modified by the simultaneous addition of pentachloronitrobenzene (PCNB) and vancomicyn was herein evaluated. The YEM base medium contained (g.l^-1^): mannitol (10.0); K_2_HPO_4 _(0.5); yeast extract (0.5); MgSO_4_.7H_2_O, (0.2); NaCl (0.007) and Congo Red (0.04). All strains were initially evaluated by their ability to grow on YEM agar media modified by the addition of PCNB, named P-YEM. This compound was initially solubilized in 98% ethanol, sterilized using 0.2 μm filters, added to 45°C YEM medium, and homogenized to obtain a final concentration of 0,2 g.l^-1 ^of PCNB into the P-YEM medium. Minimal inhibitory concentrations (MIC) of vancomycin were determined using the general protocol recommended by the Clinical and Laboratory Standards Institute (CLSI, 2008). Minimum inhibitory concentration (MIC) is defined as the lowest concentration of an antimicrobial that will inhibit the visible growth of a microorganism after overnight incubation in a defined culture medium, which in this case was modified to fit nutritional and growth characteristics of rhizobia. For that purpose, vancomycin (Laboratorios Richet S.A, Argentina) was added to the YEM medium to obtain 16, 8, 4, 2, 1, 0.5, 0.25 and 0.125 mg.l^-1 ^of antibiotic in the V-YEM medium. *B. japonicum *was inoculated at 10^4 ^cfu.tube^-1 ^by means of a Steers multinoculator (Dynatech Laboratories, Inc. Alexandria) and incubated at 30°C during 7 days. Finally, the P-YEM medium was modified by the addition of 1 mg.l^-1 ^of vancomycin to obtain the final medium, named PV-YEM.

### Model of *B. japonicum *mortality on inoculated soybean seeds

One thousand grams of soybean seed (cv. Don Mario 4870) samples previously equilibrated at 30°C were individually inoculated with 3.0 ml of stationary YEM culture medium of *B. japonicum *strain 61A273 and maintained at the same temperature during 5, 15 and 30 min; 1, 2, 3, 4, 6, 10, 18 and 24 hours until evaluation. At each selected time, bacterial counts were performed using a sub-sample of 100 seeds rinsed and soaked in 250 ml Erlenmeyer flasks containing 100 ml sterile saline solution and using a Variomag Poly magnetic stirrer 15 (Thermo Fischer Scientific, Kansas) at 450-500 rpm during 15 minutes. After that, decimal dilutions were performed in 20 ml tubes containing 9 ml of saline solution until 10^-4^. Finally, 0.1 ml of each dilution was inoculated in triplicate on YEM agar plates containing 0.2.g.l^-1 ^PCNB and 1.0 mg.l^-1 ^vancomycin (PV-YEM), according to previous results of this work. Plates were incubated in an inverted position at 30°C during 7 days. Control treatments were made to discard the possible count of non-inoculated rhizobial population contaminating the seeds. Results were expressed as the recovery factor percentage (RFP) as defined in Equation 1 of the statistical analysis section. The RFP was defined as the value resulting from the number of bacteria recovered from the seeds in relation to the theoretical number of bacteria supplied by the inoculant dose used during the seed treatment (i.e. an inoculant dose of 3 ml.kg^-1 ^soybean seeds). Bacterial cfu.ml^-1 ^from inoculants was obtained individually each time.

### Rhizobia mortality on different cultivars seeds

Soybean seed samples representing the most widely used cultivars in Argentina during 2008-2009 (Table 2) were collected from five agronomical regions of the country with the purpose of evaluating the *B. japonicum *61A273 mortality model on soybean seeds. To that end, three 1000 g samples of each cultivar were used and processed as previously described. In all cases, untreated controls were performed to discard the counting of native rhizobial population on seeds.

**Table 2:** Soybean seeds and cultivars

**Soybean cultivar **^**(1)**^	Commercial denomination	**Maturity Group **^**(2)**^	**Moisture content **^**(3)**^
4670	Don Mario	4	< 10.0
4970	Don Mario	4	< 10.0
5.1L	Don Mario	5	< 10.0
3810-pb(*)	Don Mario	4	< 10.0
4800	Don Mario	4	< 10.0
4870	Don Mario	4	< 10.0
6200	Don Mario	6	< 10.0
4990 RG	Nidera	4	< 10.0
3731 RG	Nidera	3	< 10.0
5485 RG	Nidera	5	< 10.0
NK 38-00	Syngenta	3	< 10.0
NK 48-00	Syngenta	4	< 10.0

Description of seeds used for this work and some ecophysiological, physical, commercial and microbiological characteristics

Three samples were obtained from each cultivar

^(1) ^No native rhizobia or bradyrhizobia were detected

^(2) ^Soybean maturity group describes the time from flowering to harvest maturity

^(3) ^Gravimetric determination for commercial storage recommendation (%)

^(*) ^pb (prebásica)

### Mortality of *B. japonicum *under different seed storage conditions

To evaluate the effect of variable seed storage conditions on *B. japonicum *mortality, soybean seeds of a single commercial cultivar (cv. Don Mario 4870) were inoculated with similar doses (3 ml.kg^-1^) and physiological conditions to those of the *B. japonicum *strain 61A273 culture in stationary growth phase, and maintained at three decreasing temperatures: 30°C, 20°C and 10°C during 5, 15 min, 30 min, 1 h, 2 h,3 h, 4 h, 6 h, 18 h and 24 h until evaluation. All results were informed as recovery factor percentages (RFP).

### Influence of individual components at the inoculation process

We evaluated *B. japonicum *mortality according to the variation of three agronomic parameters: (1) seed cultivars; (2) bacterial culture (inoculant) and (3) use of additive compounds during the inoculation process. For that purpose, *B. japonicum *strains 61A273 obtained from different production lots were inoculated in similar doses (3.0 ml.kg^-1^) into two commercial cultivars of soybean seeds Don Mario 4870 and 3810-pb with or without addition of 0.3 ml.kg^-1^of 80% sucrose solution as bacterial protector. All treatments are described in Table 3. Results were informed as recovery factor percentages (RFP) and direct bacterial count of *B. japonicum*.

**Table 3:** Recovery factor percentage

**soybean seeds **^(*)^	**R**_**4 **_^(**) ^**[cfu.seed**^**-1**^**]**	**R**_**0 **_^(**) ^**[cfu.seed**^**-1**^**]**	**RFP 4 hours at 20°C **^(***)^
4870 sample 1 ^(AD)^	1.90 10^5^	1.47 10^6^	12.9
4870 sample 2 ^(AD)^	1.52 10^5^	1.12 10^6^	13.6
4870 sample 3 ^(AD)^	1.53 10^5^	9.82 10^5^	15.6
3810-pb sample 1 ^(AD)^	1.40 10^5^	2.65 10^6^	5.28
3810-pb sample 1 ^(PD)^	4.00 10^5^	2.65 10^6^	15.1
3810-pb sample 2 ^(AD)^	1.20 10^5^	2.50 10^6^	4.8
3810-pb sample 2 ^(PD)^	3.90 10^5^	2.50 10^6^	15.6

Bacterial direct count and RFP at 20°C after 4 hours of inoculation with *B. japonicum *strain 61A273 into two commercial soybean seeds

R_4_: cfu.seed^-1 ^4 hours after inoculation; R_0_: cfu.seed^-1 ^theoretically provided by inoculant; RFP: Recovery Factor (%).

(*) cv. 4870: 6,200 seeds.kg^-1 ^and cv. 3810-pb: 6,000 seeds.kg^-1^

(**) average of three determinations

(***) bacterial counting in inoculant used for cv. 4870: sample 1 (3.04 10^9 ^cfu.ml^-1^); sample 2 (2.32.10^9 ^cfu.ml^-1^); sample 3 (2.03.10^9 ^cfu.ml^-1^).bacterial counting in inoculant used for cv. 3810-pb: sample 1 (5.3 10^9 ^cfu.ml^-1^); 3810-prebásica sample 2 (5.0 10^9 ^cfu.ml^-1^).

^(AD) ^treated with a dose of 3 ml.kg^-1 ^seed of the inoculant (agronomic dose)

^(PD) ^treated with a dose of 3 ml.kg^-1 ^seed of inoculant and 0.3 ml.kg^-1^of sucrose 80% solution

### Experimental design and statistical analysis

For evaluation of *B. japonicum *mortality on inoculated and commercial soybean seed experiments, three independent determinations were performed in triplicate for each treatment. For evaluation of rhizobial survival under different seed storage conditions and influence of each individual component of the inoculation process, all treatments were run in triplicate in a single experiment.

The recovery factor percentages (RFPs) were calculated using Equation 1. For the mathematical function analysis, RFPs were adjusted to a two-phase exponential decay non-linear regression function (p < 0.05) with the use of Prism 4.0 software as described by Equation 2.(1)

R_n_: number of bradyrhizobia recovered from seeds at n incubation times; T: number of theoretical bradyrhizobia provided by the inoculant(2)

Plateau: Y value at infinite times, expressed in the same units as Y; KFast and KSlow are the two rate constants, expressed in reciprocal of the X axis time units (If X is in minutes, then K is expressed in inverse minutes); Span^Fast ^and Span^Slow ^are the two time constants expressed in the same units as the X axis (They are calculated as the reciprocals of the rate constants).

## Results

### Formulation and evaluation of the selective medium

**Figure 1:**
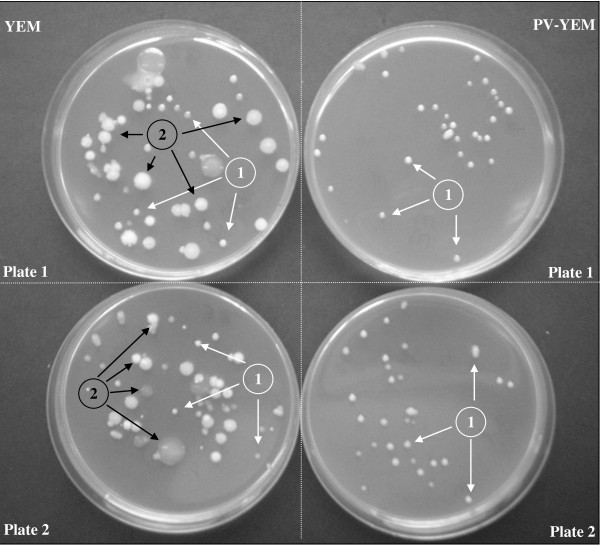
**Direct plate countof *B. japonicum***. Direct plate count of *B. japonicum *strain 61A273 recovered from non-sterilized and inoculated (dose: 3 ml.kg^-1^) soybean seeds. Photography illustrates the colony types of bradyrhizobia cells (1) and several non-bradyrhizobia cells (2) recovered from the 10^-4 ^dilution and inoculated into the YEM and PV-YEM media as described in material and methods.

Based on the vancomycin minimal inhibitory concentrations obtained for all evaluated strains summarized in Table 1, it was decided to add 1 mg.l^-1 ^of vancomycin to the P-YEM medium. Considering the whole population of samples analyzed during the experiments, which were mostly obtained from different soybean cultivars and regions of Argentina, it was found that the PV-YEM medium showed ability to prevent the growth of Gram-positive microbiota and fungi when compared with the YEM base media, and this fact improved the efficiency of bradyrhizobial count on soybean seeds. There were no significant differences in relation to *B. japonicum's *ability to grow on PV-YEM agar plates, (5.4; 5.1 and 5.2 10^9 ^cfu.ml^-1^) compared with the YEM base media (5.3 10^9 ^cfu.ml^-1^). To illustrate this fact, Figure 1 shows the presence of *B. japonicum *cells recovered from soybean seeds by using the PV-YEM medium, as well as the inhibition of soybean seed microbiota when using the YEM base formulation.

### *B. japonicum *survival on inoculated soybean seeds

**Figure 2:**
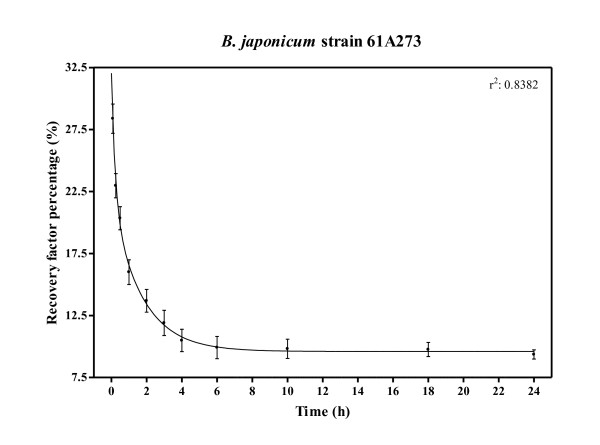
**Mortality of *B. japonicum***. Exponential decay function generated with recovery factor percentage (RFP) at increasing incubation time after inoculation with *B. japonicum *strain 61A273 on commercial soybean seeds (Table 2), previously equilibrated at 30°C. Values shown are mean ± SD.

Recovery factors (RFP) obtained by the inoculation of *B. japonicum *strain 61A273 into commercial soybean seeds (Table 2) are described in Figure 2. The number of recovered bradyrhizobia measured as RFPs decayed abruptly from an average of 29% to 10%, until the start of a plateau 4 hours after inoculation (Figure 2). The RFP values obtained before the plateau were very variable and absolutely dependent on each sample processing time, with high variations in a short period of time. After 4-6 hours and until 24 hours, the percentage of recovered cells was stable and only presented minor variations. In our experiments, RFP was around 10%.

### *B. japonicum *recovery and survival under different seed storage conditions

**Figure 3:**
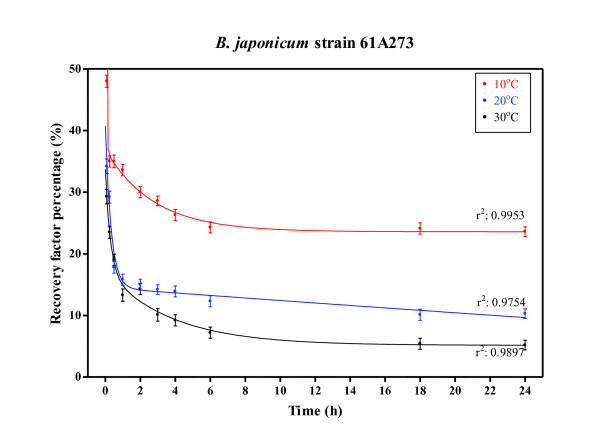
**Temperature effect on *B. japonicum *mortality**. Exponential decay function of recovery factor percentage (RFP) at increasing incubation times and at incubation temperatures of 10°C, 20°C and 30°C, after inoculation with *B. japonicum *strain 61A273 on soybean seeds cv. Don Mario 4870. Values shown are mean ± SD.

In our experiments, as a result of the temperature range from 10°C to 30°C (that may represent extreme inoculation conditions, ranging from the low temperatures at which seeds are exposed in Canada to late planting time in more template regions) at planting time, the number of recovered bradyrhizobia as RFP at 4-6 hours after inoculation decayed abruptly at 30°C rather than at 20°C or 10°C, respectively (Figure 3). The high incubation temperature stabilized at 9.2% at 4 hours and finally reached the minimal of 5.2% at 18 hours. At 20°C, it stabilized at 13.4% and reached the minimal at 10.2%. Finally, at 10°C, stabilization was reached at 26% and the minimal value at 23.6%.

### Influence of individual components at the inoculation process

Table 3 shows the versatility of the proposed protocol under several experimental conditions. During evaluation of the same inoculant and seed samples, exemplified by cv. 4870, no significant differences were obtained at RFPs. On the other hand, compared with cv. 4870, the influence of different seed cultivars, exemplified by cv. 3810-pb, but treated with the same inoculant, significantly modified the RFPs. Comparisons could be made by the addition of an 80% sucrose (w/v) solution together with the seed inoculant. In this case, the addition of the sucrose solution reverted, at least partially, the mortality of *B. japonicum *on seeds, measured as RFPs during the first 4 hours after inoculation.

## Discussion

The inoculation of plants with beneficial bacteria can be traced back for centuries (Bashan, 1998). By the end of the 19th century, the practice of mixing "naturally inoculated" soil with seeds became a recommended method of legume inoculation in the USA (Smith, 1992). A decade later, the first patent for plant inoculation with *Rhizobium *sp was registered. (Nobbe and Hiltner, 1896). The practice of soybean inoculation with *Bradyrhizobium *sp. became common and economically recommended in many producer countries. In Argentina and many South American countries, soybeans [*Glycine max *(Merr.) L.] are commonly not fertilized but only inoculated with nitrogen. In 2010, Argentina, Bolivia, Paraguay and Uruguay produced more than 20 million hectares of soybeans, almost 16 million of which (more than 80%) were inoculated with products generated by more than 100 companies with common market.

Bacterial counts on non-selective media are a routine quality control procedure for defining a basic threshold in inoculant quality control. Those inoculants that do not fulfill the requested bacterial numbers are discarded in compliance with different regulations (country dependent). These counts are easily performed, even by small, non-specialized microbiology laboratories when inoculants only contain the desired rhizobial population, that is, when inoculants are formulated in a sterile carrier. However, most inoculants are finally applied on non-sterile material like seeds or even directly on the soil. Once there, rhizobial enumeration is a non-reproducible task, due to the presence of Gram-positive bacteria and fungi in variable numbers that may interfere with their direct growth, as in the case of fast growers, or by the synthesis and secretion of toxic compounds, which make comparisons among different formulations for on-seed survival very difficult to achieve, thus delaying better formulation developments.

Some selective agents have been previously used for selective enumeration of *Rhizobium *and *Bradyrhizobium *sp. from soils and non-sterile inoculants. Selective agents included antibiotics, heavy metals, dyes and metabolic inhibitors (Danso et al, 1973; Graham, 1969; Thompson, 1967; Van Schreven, 1970). Pattison and Skinner (1974) reported a formulation that contained pentachloronitrobenzene (PCNB), brilliant green (BG), sodium azide, crystal violet, and penicillin. Gómez et al. (1995) proposed two selective media for the enumeration of *B. japonicum *from soybean inoculants (at that time mostly using non-sterile peat as carrier) containing tetracycline, rifampicin and chloramphenicol to control bacteria and cycloheximide and pimafucin to control fungal contaminants. In our study, most fungal contamination could be prevented by using previously reported PCNB concentrations on the YEM base medium (P-YEM). However, the presence of large mucoid colonies of some gram-positive bacilli after one week incubation prevented in most cases the differentiation and enumeration of typical rhizobium colonies. Our use of the combined PV-YEM medium allowed a significant improvement in the efficiency of on-seed rhizobial enumeration as it effectively prevented the growth of Gram-positive bacteria and fungi (Figure 1).

As already stated, one of the major problems following soybean inoculation is fast bacterial death (Salema et al., 1982; Streeter, 2003). The seed storage temperature after inoculation is empirically considered the most important parameter related to rhizobial survival after seed treatment (Vriezen et al., 2005, 2006; Kremer and Peterson 1983). Moreover, temperature also directly affects the inoculated population desiccation rate generating a second stress condition (Streeter 2003 and 2007). Salinity and seed coat toxicity stress (Deaker et al., 2004) have a strong influence on bacterial survival; however, temperature and the consequent desiccation rate have been the major predicted cause of bacteria injury on seeds. Nevertheless, only few studies have involved the actual analysis of seed survival, perhaps due to contamination problems (Vincent et al., 1962; Bushby and Marshall, 1977; Jansen van Rensburg and Strijdom, 1980; Mary et al., 1986; Cliquet and Catroux, 2001, Kosanke et al., 1992; Vriezen et al., 2006). According to our results, seed storage after inoculation should be recommended at a temperature below 30°C and preferably below 20°C. We propose a survival factor percentage (SFP) defined as a single time parameter resulting from the number of bacteria recovered from seeds after the mortality function reached the plateau for decay, which in some way means when the survival bacterial population stabilized on seeds after inoculation. In our experiments, SFPs were defined 4 hours after inoculation. However, for research and development purposes, we suggest standardizing SFPs at 30°C, as these may reduce the time required for product (or product variation) analysis.

Any plant growth promoting bacteria (PGPB), including rhizobial survival on the seed surface, is usually lower than on solid carriers such as peat, vermiculite or alginate (Bashan et al., 2002) due to the lack of protection against desiccation, and/or toxic compounds present on the seed coat (Burton 1972, 1968). Even if an increase of provided *B. japonicum *cells improves the nodulation process, plant nitrogen assimilation and grain yield in laboratory conditions (Papakosta, 1992), bacterial physiological state and its resistance to environmental stress may also be critical for its survival on seed and field conditions (Streeter 2007). Roughley et al. (1993) reported a high mortality of *Bradyrhizobium *sp. after the inoculation of lupine seeds, decreasing by a factor of 10 after one hour, of one hundred after four hours and more than one thousand after one day on the seeds. Similar results were presented by Catroux et al. (1996) in *B. japonicum *inoculated soybean seeds. The microorganism population recovery from inoculated soybean seeds (as a whole) may be extremely heterogeneous when expressed as cfu.seed^-1^, depending on the two components, inoculants and seeds. In the case of inoculants, the major variation sources are the formulation, the presence of some osmoprotectant (e.g. sucrose or carboximethylcellulose), the number of viable microorganisms per volume unit and the recommended doses. In the case of seeds, normally the genotype, the size and the total surface of each grain, storage conditions, a_w_, integrity of teguments and mechanical damage can be easily mentioned. Due to all these complex variables, comparison of inoculation treatments (that is, a stated seed A with a stated inoculant A vs another seed B with an inoculant B combination) is frequently misleading.

Our use of relative values like RFPs or SFPs helps dissect the simplest questions, keeping others under the control state, and may even help compare experiments separated in time (Table 3, Figure 2).

The primary benefit of bacterial biological nitrogen (N_2_) fixation in grain and forage legumes is the enhancement of yield potentials decreasing the use of exogenous fertilizers. This ability had resulted in commercial legume inoculant production facilities worldwide (Brockwell and Bottomley, 1995). Unfortunately, the widespread availability of legume and other related inoculants had not resulted efficiently equal at level of development of quality control standards protocols. Many countries, including Australia, the Netherlands, Rwanda, Thailand and Russia (Smith, 1992; Marufu et al., 1995) have legislated requirements for minimum populations of target organisms and contaminants per weight unit of inoculants. Canada and France have legislated on product quality by defining the number of viable *Rhizobium/Bradyrhizobium *required (provided) per seed, or a rate of application, for a full range of crops. The United States and United Kingdom leave the issue on product quality and rates of application at the manufacturer's discretion (Smith, 1992). In Argentina, Brazil and other South American countries actual regulations are insufficient to put pressure on local companies to improve the quality of their products. The lack of internationally accepted regulations for legume inoculant quality and usage parameters has led, on many occasions to inadequate inoculant performance and to the subsequent abandonment of their use. To reverse this trend, a complementary method for soybean inoculant quality control was herein proposed. We improved a selective media for the enumeration of *B. japonicum *from inoculated soybean seeds, established the most representative mathematical function for *B. japonicum *mortality on soybean seeds after inoculation and generated Recovery and Survival Factor Percentages as novel tools for inoculant quality control.

## Abbreviations

MPN: most probable number; CFU: colony forming units; YEM: Yeast Extract-Mannitol; PCNB: pentachloronitrobenzene; MIC: minimal inhibitory concentration; RFP: recovery factor percentage; SFP: survival factor percentage; PGPB: plant growth promoting bacteria

## Competing interests

The authors declare that they have no competing interests.
