# A simple method to evaluate the number of bradyrhizobia on soybean seeds and its implication on inoculant quality control

**DOI:** 10.1186/2191-0855-1-25

**Published:** 2011-09-01

**Authors:** Claudio Penna, Rosana Massa, Florencia Olivieri, Gabriel Gutkind, Fabricio Cassán

**Affiliations:** 1 Laboratorio de Investigación y Desarrollo Básico. Merck Crop Bioscience Argentina. Buenos Aires, Argentina; 2 Departamento de Microbiología, Inmunología y Biotecnología, Facultad de Farmacia y Bioquímica, Universidad de Buenos Aires, Argentina; 3 Laboratorio de Fisiología Vegetal, Universidad Nacional de Rio Cuarto, Córdoba, Argentina

## Correction

The authors regret that an error appears in Penna etal. (2011). The fourth sentence in the section *Formulation and evaluation of the selective medium *"This compound was initially solubilized in 98% ethanol, sterilized using 0.2 μm filters, added to 45°C YEM medium, and homogenized to obtain a final concentration of 0,2 g.l-1 of PCNB into the P-YEM medium". Must be replaced by the following "A solution of 10% PCNB in acetone, was diluted 1/5 in Tween 80 0,5%v/v in water, sterilized using 0.2 μm filters, added to 45°C YEM medium, and homogenized to obtain a final concentration of 0,04 g.l^-1 ^of PCNB into the P-YEM medium".
